# Supplementary figures and images for: INT reduction is a valid proxy for eukaryotic plankton respiration despite the inherent toxicity of INT and differences in cell wall structure
Source: PLoS One. 2019 Dec 10;14(12):e0225954. doi: 10.1371/journal.pone.0225954 (PMC6903736; doi:10.1371/journal.pone.0225954)

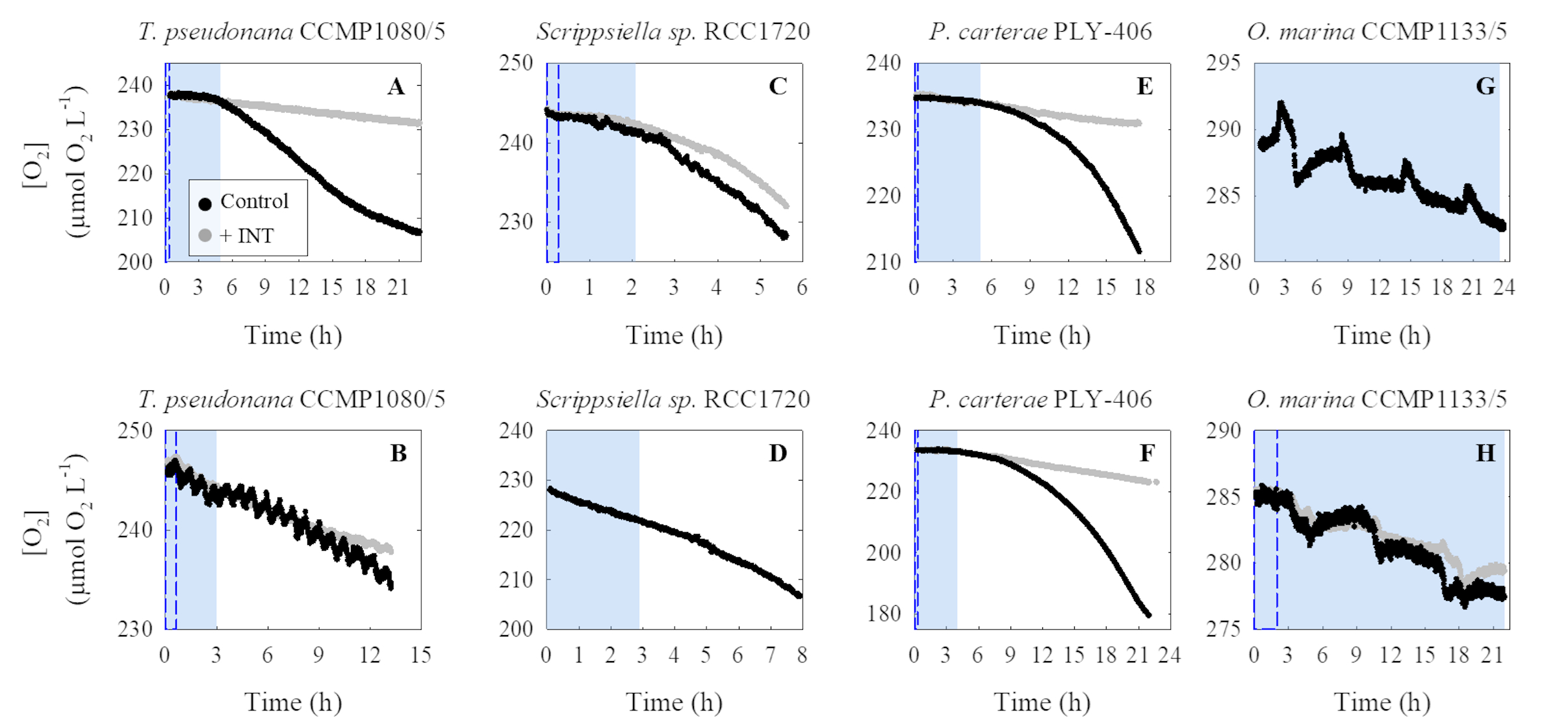

Supplement: S1 Fig — Oxygen concentration over time measured in dark incubations of Thalassiosira pseudonana CCMP1080/5 (A, B), Scrippsiella sp. RCC1720 (C, D), Pleurochrysis carterae PLY-406 (E, F) and Oxyrrhis marina CCMP1133/5 (G, H) cultures during the CRO2-INTR experiments. The oxygen concentration of samples without INT (controls) is shown in black, and that of samples after addition of 0.2 mM INT in grey. Plots D and G do not have samples with INT added due to optode sensor failure. The incubation time for the oxygen incubations measured with Winkler titrations is shown as a light blue shaded area and the incubation time for the INT reduction method as a blue dashed box. Note the linear trend in the oxygen consumption during the incubation time for the Winkler discrete samples, and the lack of any difference between the oxygen consumption with and without addition of INT. Note that the oxygen concentrations and time intervals are different for each culture experiment. (TIF) [file pone.0225954.s001.tif]
